# Hemolysis of Human Erythrocytes by Methicillin-Resistant *Staphylococcus aureus* Is Primarily Caused by PSMα Peptides

**DOI:** 10.3390/toxins17110529

**Published:** 2025-10-29

**Authors:** Tyler K. Nygaard, Annika Gao, Eliot LaTray, Jovanka M. Voyich

**Affiliations:** Department of Microbiology & Cell Biology, Montana State University, Bozeman, MT 59715, USA; annikagao3@gmail.com (A.G.); eliotlatray@gmail.com (E.L.); jovanka@montana.edu (J.M.V.)

**Keywords:** *Staphylococcus aureus*, hemolysis, human erythrocytes, PSMα, USA300, USA100, cytotoxins, leukocidins

## Abstract

*Staphylococcus aureus* (*S. aureus*) is a major cause of human morbidity and mortality worldwide. Hemolysis caused by *S. aureus* cytotoxins is important for the acquisition of iron and subsequent bacterial survival during infection. *S. aureus* can express numerous hemolysins that have been shown to target human erythrocytes. However, the relative importance of each of these for causing hemolysis during pathogenesis in humans is not clear. In this study, we have examined the hemolytic capacity of different methicillin-resistant *S. aureus* (MRSA) deletion mutants against human erythrocytes in suspension using two separate assays. The first assay measured hemolysis caused by extracellular factors produced by MRSA, while the second measured hemolysis following co-culture of MRSA with human erythrocytes. Results from both assays demonstrated that phenol-soluble modulin-α peptides (PSMα) play a dominant role in causing hemolysis of human erythrocytes, highlighting a prominent target for novel therapeutic strategies designed to limit *S. aureus* iron acquisition and survival during human disease.

## 1. Introduction

*Staphylococcus aureus* (*S. aureus*) is a common Gram-positive bacterium that can cause a wide variety of infections in humans and animals, ranging from superficial skin abscesses to systemic life-threatening disease [1,2]. The ability of *S. aureus* to infect different host tissues is primarily attributed to a diverse array of virulence factors expressed by this pathogen. These include numerous host immunomodulatory proteins, adhesins, and cytotoxins. The large number and apparent redundancy of these virulence factors have made it difficult to determine the relative contribution of each towards different aspects of *S. aureus* pathogenesis. To complicate matters further, the specificity of many of these virulence factors depends upon the species of host organism [3,4,5,6,7,8,9,10,11,12,13,14,15]. This limits the translation of experimental results using non-human cell types and animal models of infection to understand *S. aureus* pathogenesis in humans.

*S. aureus* expresses an arsenal of cytotoxins that each have varying degrees of specificity against different cell types and between different host organisms [16]. Some of these are highly conserved between different strains of *S. aureus*, such as α-toxin (Hla) and the phenol-soluble modulin peptides (PSMs). Others are less conserved and are part of the accessory *S. aureus* genome, such as the Panton–Valentine leukocidin (PVL). The *S. aureus* strain identified by pulsed-field gel electrophoresis (PFGE) as USA300 is a dominant clinical isolate in the United States and expresses at least thirteen different cytotoxins that include Hla, PVL, leukocidin E/D (LukED), γ-hemolysin AB (HlgAB), γ-hemolysin CB (HlgCB), leukocidin G/H (LukGH [17], also known as LukAB [18]), as well as the family of PSMs that consist of δ-toxin (Hld), four phenol-soluble modulin-α peptides (PSMα) encoded on a single operon, and two phenol-soluble modulin-β peptides (PSMβ) also encoded on a single operon. The upregulation of these cytotoxins during infection is thought to be largely mediated by the accessory gene regulator (Agr) two-component system and the *S. aureus* exoprotein (Sae) two-component system [19,20]. Activation of both of these gene regulatory systems is required to increase expression of almost all *S. aureus* cytotoxins. Notable exceptions are the PSMs that are regulated by Agr [21,22] but not by Sae [23,24].

Iron is essential for metabolic processes in almost all organisms, and the acquisition of iron by *S. aureus* in the host is important for pathogenesis [25]. *S. aureus* obtains iron primarily from heme released by erythrocytes following cytotoxin-dependent hemolysis using the iron-regulated surface determinant (Isd) system [26,27]. Thus, clearly defining important *S. aureus* cytotoxins that target human erythrocytes will highlight factors that further iron acquisition to allow bacterial survival during infection. The majority of research investigating *S. aureus* hemolysis has used sheep erythrocytes in agar or rabbit erythrocytes in suspension to determine the cytotoxins that are responsible. However, there are major differences in the specificity of *S. aureus* cytotoxins against human erythrocytes compared to sheep or rabbit erythrocytes [9,11,12], and agar has been reported to inhibit the hemolytic activity of HlgAB [11].

Compared to hemolysis of sheep or rabbit erythrocytes, there are relatively few studies that have examined hemolysis of human erythrocytes by *S. aureus* cytotoxins. These have indicated that HlgAB, LukED, and PSMs play important roles in this process [28,29,30,31,32], but the lack of studies that directly compare the hemolytic activity of each of these cytotoxins has limited our understanding of which is the most important for causing hemolysis during pathogenesis in humans. In this investigation, we have used a library of *S. aureus* cytotoxin deletion mutants in two different ex vivo hemolysis assays to define the relative susceptibility profile of human erythrocytes to *S. aureus* cytotoxins. Findings in this report demonstrate that the PSMα peptides play a dominant role in the hemolysis of human erythrocytes by *S. aureus*.

## 2. Results

### 2.1. Hla Is Important for Hemolysis of Sheep Erythrocytes but Not Human Erythrocytes

**Figure 1:**
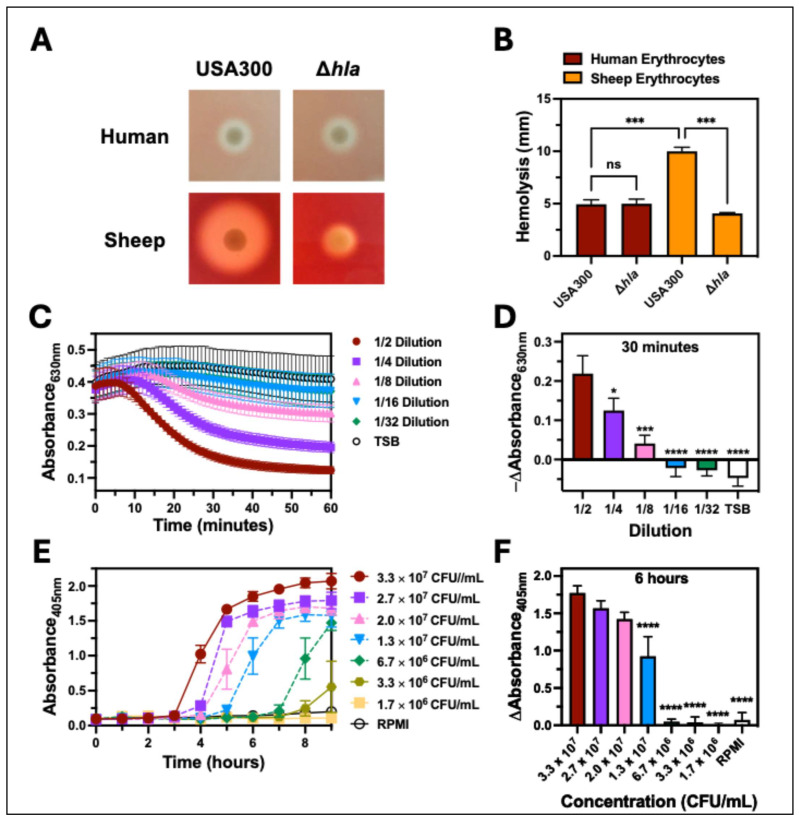
Hemolysis of human erythrocytes by USA300. (**A**) Representative colonies and (**B**) hemolysis diameter of USA300 and an isogenic deletion mutant of *hla* in USA300(Δ*hla*) on TSA agar with human or sheep erythrocytes. (**C**) Turbidity of suspended human erythrocytes was measured by absorbance at 630 nm following intoxication with USA300 supernatants at indicated dilutions for 60 min at 37 °C. (**D**) The change in absorbance at 630 nm from data collected in panel C determined after 30 min of intoxication. (**E**) Extracellular heme was measured by absorbance at 405 nm of supernatants from human erythrocytes co-cultured with indicated concentrations of USA300 and incubated at 37 °C for 9 h. (**F**) The change in absorbance at 405 nm from data collected in panel (**E**) determined at 6 h post-infection. All data are the mean ± SEM of at least 3 independent experiments with * *p* ≤ 0.05, *** *p* ≤ 0.001, and **** *p* ≤ 0.0001 relative to USA300, as determined by repeated-measures one-way ANOVA with Tukey’s multiple comparison test for panel (**B**) or Dunnett’s multiple comparison test for panels (**D**,**F**).

To initially examine hemolysis caused by *S. aureus*, we compared the hemolytic activity of the USA300 wild type (USA300 wt) relative to an isogenic deletion mutant of *hla* in USA300 (Δ*hla*) against sheep erythrocytes and human erythrocytes in tryptic soy agar (Figure 1A). The zone of hemolysis produced by USA300 was significantly larger against sheep erythrocytes relative to human erythrocytes (Figure 1B). In addition, a significant decrease in the hemolysis of sheep erythrocytes caused by Δ*hla* relative to USA300 was not observed with human erythrocytes (Figure 1B). These findings indicate that the specificity of hemolytic factors produced by *S. aureus* varies between host species and that hemolysis of human erythrocytes was primarily caused by factors other than Hla, corresponding to previously published findings [3,33].

Erythrocytes in agar do not accurately represent in vivo conditions encountered by *S. aureus* during infection, and others have indicated that the hemolytic activity of HlgAB is limited on agar [11]. To more appropriately address hemolysis caused by *S. aureus* during human pathogenesis, we employed two distinct assays that examine hemolysis of human erythrocytes in suspension. The first assay used extracellular factors produced by *S. aureus* cultures at early stationary growth to intoxicate human erythrocytes in suspension and assessed changes in turbidity that occurred following hemolysis by measuring absorbance at 630 nm over time (Figure 1B,C). The second assay used *S. aureus* harvested at mid-exponential growth to infect human erythrocytes in suspension and assessed changes in extracellular heme released following hemolysis by measuring the absorbance of heme at 405 nm of supernatants from infected samples (Figure 1D,E).

### 2.2. The Agr Two-Component System Is Required for Hemolysis of Human Erythrocytes

**Figure 2:**
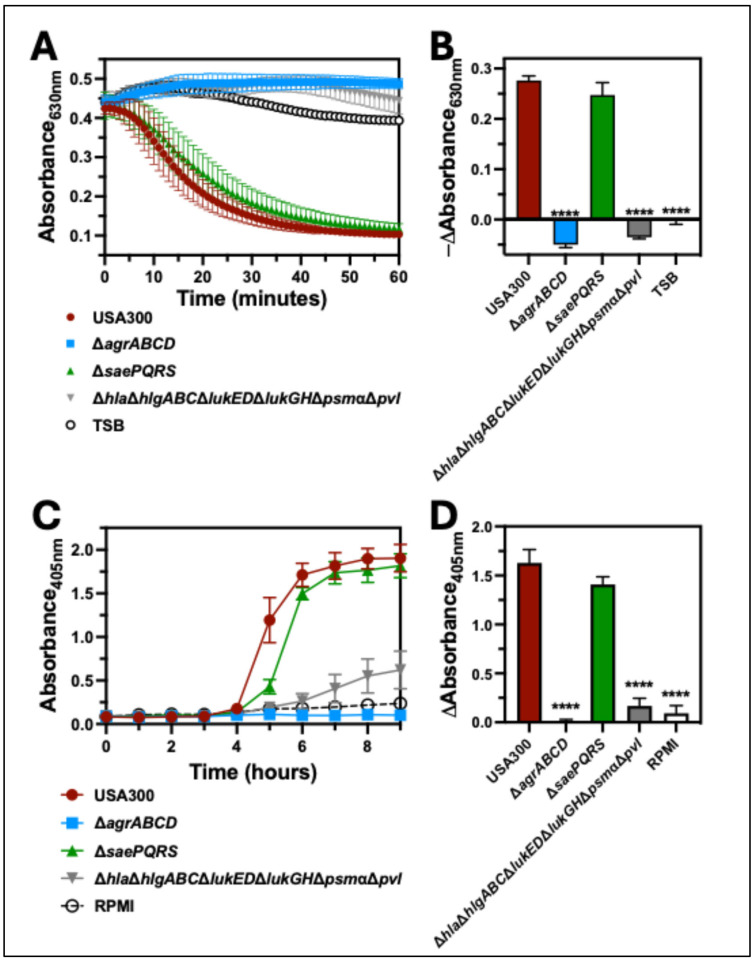
Hemolysis of human erythrocytes by USA300 is dependent upon the Agr two-component system. (**A**) Turbidity of suspended human erythrocytes was measured by absorbance at 630 nm following intoxication with supernatants from cultures of USA300, USA300 lacking the *agr* two-component system (Δ*agrABCD*), USA300 lacking the *sae* two-component system (Δ*saePQRS*), or USA300 lacking *hla*, *hlgABC*, *lukED*, *lukGH*, *psmα*, and *pvl* (Δ*hla*Δ*hlgABC*Δ*lukED*Δ*lukGH*Δ*psm*αΔ*pvl*) for 60 min at 37 °C. (**B**) The change in absorbance at 630 nm from data collected in panel A determined after 30 min of intoxication. (**C**) Extracellular heme was measured by absorbance at 405 nm of supernatants from human erythrocytes co-cultured with USA300, Δ*agrABCD*, Δ*saePQRS*, or Δ*hla*Δ*hlgABC*Δ*lukED*Δ*lukGH*Δ*psmα*Δ*pvl* and incubated at 37 °C for 9 h. (**D**) The change of absorbance at 405 nm from data collected in panel C determined at 6 h post-infection. All data are the mean ± SEM of at least 3 independent experiments with **** *p* ≤ 0.0001 relative to USA300 as determined by repeated-measures one-way ANOVA with Dunnett’s multiple comparison test.

Both Agr and Sae play important roles in mediating the upregulation of cytotoxin expression by *S. aureus*. To verify the importance of these two-component systems for hemolysis of human erythrocytes, we first compared the hemolytic capacity of extracellular factors produced by USA300 wt, a USA300 deletion mutant of *agrABCD* (Δ*agr*), a USA300 deletion mutant of *saePQRS* (Δ*sae*), and a USA300 deletion mutant of the cytotoxins *hla*, *hlgABC*, *lukED*, *lukGH*, *psm*α, and *PVL* (Δ*hla*Δ*hlgABC*Δ*lukED*Δ*lukGH*Δ*psm*αΔ*pvl*) against human erythrocytes (Figure 2A). As expected, the turbidity of erythrocytes in solution following intoxication with supernatants from USA300 wt was significantly decreased relative to intoxication with supernatants from Δ*agr* or Δ*hla*Δ*hlgABC*Δ*lukED*Δ*lukGH*Δ*psm*αΔ*pvl* (Figure 2B). Surprisingly, we found that extracellular factors produced by Δ*sae* only caused a delayed drop in the turbidity of intoxicated erythrocytes relative to USA300 wt (Figure 2A) with no significant difference noted at 30 min after intoxication (Figure 2B). Similar observations were made when human erythrocytes were infected with USA300, Δ*agr*, Δ*sae*, or Δ*hla*Δ*hlgABC*Δ*lukED*Δ*lukGH*Δ*psm*αΔ*pvl* (Figure 2C); an increased absorbance of released heme at 405 nm over time following inoculation with USA300 wt was only slightly delayed following inoculation with Δ*sae* (Figure 2C), while inoculation with Δ*agr* or Δ*hla*Δ*hlgABC*Δ*lukED*Δ*lukGH*Δ*psm*αΔpvl produced significantly lower absorbance at 405 nm relative to USA300 wt (Figure 2D). Together, these findings suggested that cytotoxins regulated by Agr play a prominent role in the hemolysis of human erythrocytes, while those regulated by Sae are less important.

### 2.3. Bicomponent Leukocidins Are Not Necessary for Hemolysis of Human Erythrocytes

**Figure 3:**
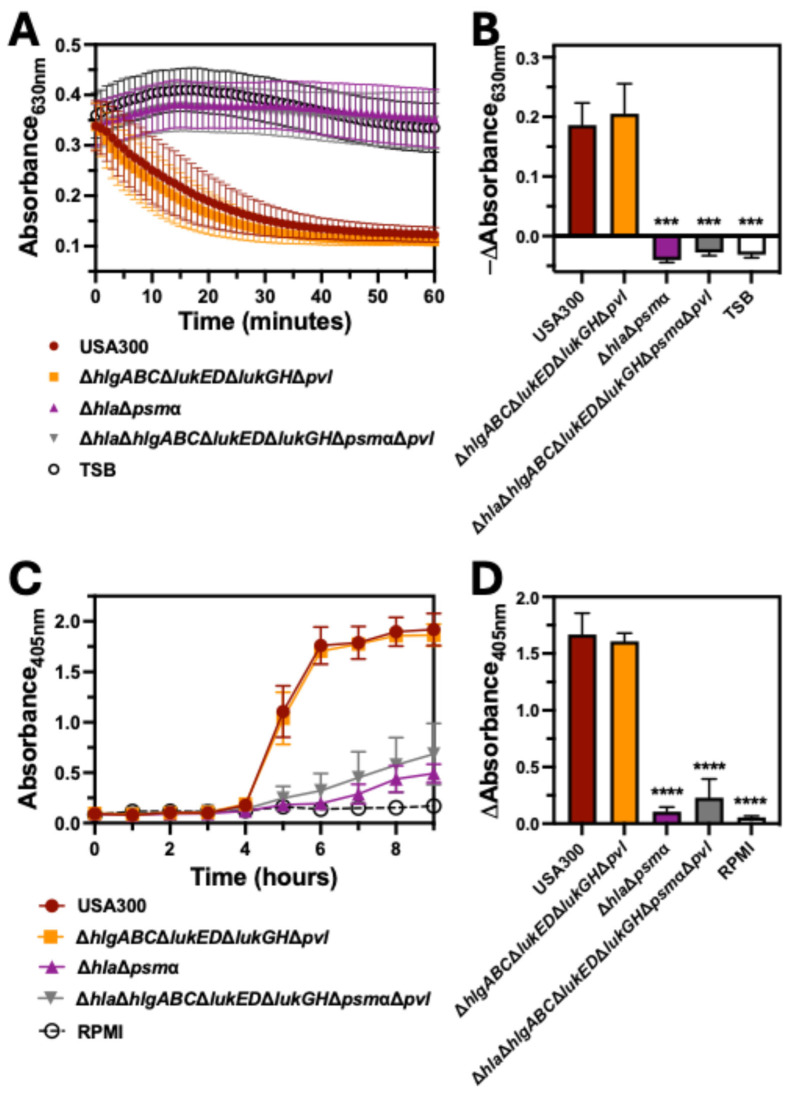
Bicomponent leukocidins are not required for hemolysis of human erythrocytes by USA300. (**A**) Turbidity of suspended human erythrocytes was measured by absorbance at 630 nm following intoxication with supernatants from cultures of USA300, USA300 lacking *higABC*, *lukED*, *lukGH*, and *pvl* (Δ*hlgABC*Δ*lukED*Δ*lukGH*Δ*pvt*), USA300 lacking *hla* and *psmα* (Δ*hla*Δ*psmα*), or USA300 lacking *hla*, *hlgABC*, *lukED*, *lukGH*, *psmα*, and *pvl* (Δ*hla*Δ*hlgABC*Δ*lukED*Δ*lukGH*Δ*psmα*Δ*pvl*) for 60 min at 37 °C. (**B**) The change in absorbance at 630 nm from data collected in panel A determined after 30 min of intoxication. (**C**) Extracellular heme was measured by absorbance at 405 nm of supernatants from human erythrocytes co-cultured with USA300, Δ*hlgABC*Δ*lukED*Δ*lukGH*Δ*pvl*, Δ*hla*Δ*psmα*, or Δ*hla*Δ*hlgABC*Δ*lukED*Δ*lukGH*Δ*psmα*Δ*pvl* and incubated at 37 °C for 9 h. (**D**) The change of absorbance at 405 nm from data collected in panel C determined at 6 h post-infection. All data are the mean ± SEM of at least 3 independent experiments with *** *p* ≤ 0.001 and **** *p* ≤ 0.0001 relative to USA300, as determined by repeated-measures one-way ANOVA with Dunnett’s multiple comparison test.

The observed hemolytic activity of Δ*sae* suggested that the bicomponent leukocidins HlgAB, HlgCB, LukED, LukGH, and PVL, which are all transcriptionally regulated by this two-component system, are dispensable for hemolysis caused by USA300. To test this, we examined hemolysis caused by a USA300 deletion mutant of *hlgABC*, *lukED*, *lukGH*, and *pvl* (Δ*hlgABC*Δ*lukED*Δ*lukGH*Δ*pvl*), a USA300 deletion mutant of *hla* and *psm*α (Δ*hla*Δ*psm*α), and USA300 lacking all of these cytotoxins (Δ*hla*Δ*hlgABC*Δ*lukED*Δ*lukGH*Δ*psm*αΔ*pvl*). Intoxication of human erythrocytes with extracellular factors produced by Δ*hlgABC*Δ*lukED*Δ*lukGH*Δ*pvl* caused a decrease in the absorbance at 630 nm over time that paralleled intoxication with extracellular factors produced by USA300 wt (Figure 3A). In contrast, extracellular factors produced by USA300 deletion mutants lacking *hla* and *psm*α (Δ*hla*Δ*psm*α and Δ*hla*Δ*hlgABC*Δ*lukED*Δ*lukGH*Δ*psm*αΔ*pvl*) caused significantly less change in absorbance at 630 nm relative to USA300 wt (Figure 3B). As with intoxication assays, inoculation of human erythrocytes with Δ*hla*Δ*psm*α and Δ*hla*Δ*hlgABC*Δ*lukED*Δ*lukGH*Δ*psm*αΔ*pvl* resulted in significantly less absorbance of released heme at 405 nm over time relative to inoculation with USA300 wt or Δ*hlgABC*Δ*lukED*Δ*lukGH*Δ*pvl* (Figure 3C,D). These findings indicated that hemolysis caused by USA300 was independent of the bicomponent leukocidins but did require Hla and/or PSMα.

### 2.4. PSMα Is the Major Hemolytic Factor Produced by S. aureus Against Human Erythrocytes

**Figure 4:**
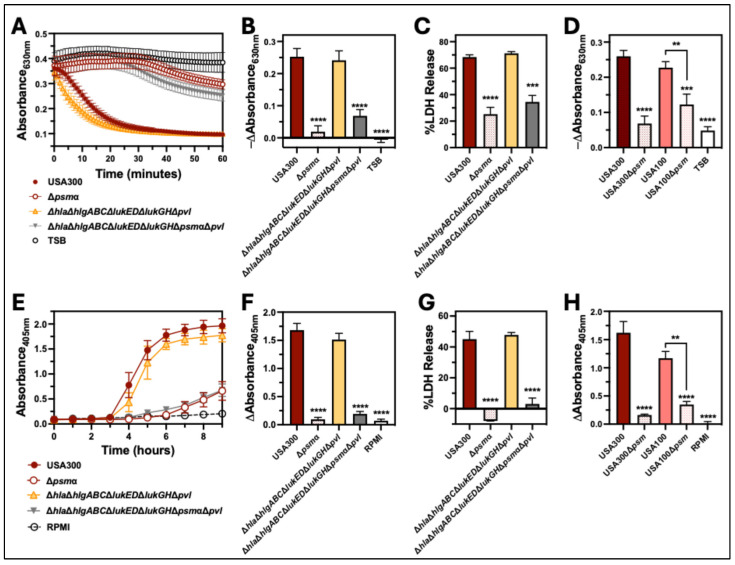
Deletion of PSM-α significantly reduces hemolysis of human erythrocytes caused by *S. aureus.* (**A**) Turbidity of suspended human erythrocytes was measured by absorbance at 630 nm following intoxication with supernatants from cultures of USA300, USA300 lacking *psmα* (Δ*psmα*), USA300 lacking *hla*, *hlgABC*, *lukED*, *lukGH*, and *pvl* (Δ*hla*Δ*hlgABC*Δ*lukED*Δ*lukGH*Δ*pvl*), or USA300 lacking *hla*, *hlgABC*, *lukED*, *lukGH*, *psmα*, and *pvl* (Δ*hla*Δ*hlgABC*Δ*lukED*Δ*lukGH*Δ*psmα*Δ*pvl*) for 60 min at 37 °C. (**B**) The change in absorbance at 630 nm from data collected in panel (**A**) after 30 min and (**C**) lactate dehydrogenase release after 40 min of intoxication. (**D**) Change in absorbance at 630 nm following intoxication with 8 h supernatants from USA300, USA100, and corresponding deletion mutants of *psmα* in each strain (USA300Δ*psmα* and USA100Δ*psmα*, respectively) after 30 min of intoxication. (**E**) Extracellular heme was measured by absorbance at 405 nm of supernatants from human erythrocytes co-cultured with USA300, Δ*psmα*, Δ*hla*Δ*hlgABC*Δ*lukED*Δ*lukGH*Δ*pvl*, or Δ*hla*Δ*hlgABC*Δ*lukED*Δ*LukGH*Δ*psmα*Δ*pvl* and incubated at 37 °C for 9 h. (**F**) The change in absorbance at 405 nm from data collected in panel (**E**). (**G**) Lactate dehydrogenase release determined 6 h after co-culture with indicated *S. aureus* strains. (**H**) Change in absorbance at 405 of supernatants from human erythrocytes co-cultured with USA300, USA300Δ*psmα*, USA100, and USA100Δ*psmα* for 6 h. All data are the mean ± SEM of at least 3 independent experiments with ** *p* ≤ 0.01, *** *p* ≤ 0.001, and **** *p* ≤ 0.0001 relative to USA300, as determined by repeated-measures one-way ANOVA with Dunnett’s multiple comparison test panels (**A**–**C**,**E**–**G**) or Tukey’s multiple comparison test panels (**D**,**H**).

**Figure 5:**
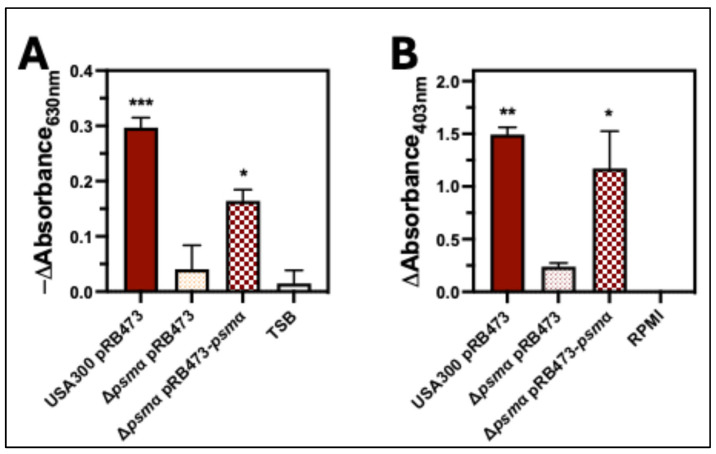
Complementation of USA300Δpsmα with pRB473-psmα rescues hemolysis. (**A**) The change in the absorbance at 630 nm of human erythrocytes intoxicated for 60 min with 8 h supernatants from USA300 transformed with pRB473 (USA300 pRB473), USA300 lacking *psmα* transformed with pRB473 (Δ*psmα* pRB473), USA300 lacking *psmα* transformed with pRB473 encoding *psmα* (Δ*psmα* pRB473-*psmα*), or TSB control. (**B**) Change in absorbance at 405 nm of supernatants from human erythrocytes infected for 7 h with USA300 pRB473, Δ*psmα* pRB473, Δ*psmα* pRB473-*psmα*, or RPMI. All data are the mean ± SEM of at least 3 independent experiments with * *p* ≤ 0.05, ** *p* ≤ 0.01, and *** *p* ≤ 0.001 relative to Δ*psmα* pRB473, as determined by repeated-measures one-way ANOVA with Dunnett’s multiple comparison test.

Our results showed that Δ*agr* and Δ*hla*Δ*psm*α are defective in causing hemolysis of human erythrocytes, while hemolysis caused by Δ*sae* was only slightly delayed relative to USA300 wt. Both Agr and Sae upregulate expression of Hla, but expression of PSMα is only controlled by Agr [21,22] and not Sae [23,24], suggesting that PSMα is primarily responsible for causing hemolysis of human erythrocytes. To test this, we examined the hemolytic capacity of an isogenic deletion mutant of *psm*α in USA300 (Δ*psm*α*)* compared to a deletion mutant of cytotoxins other than *psm*α in USA300 (Δ*hla*Δ*hlgABC*Δ*lukED*Δ*lukGH*Δ*pvl*) (Figure 4). Intoxication of human erythrocytes with extracellular factors produced by mutants lacking *psm*α (Δ*psm*α and Δ*hla*Δ*hlgABC*Δ*lukED*Δ*lukGH*Δ*psm*αΔ*pvl*) resulted in a significant decrease in the change in absorbance at 630 nm and release of lactate dehydrogenase (LDH) relative to USA300 wt that was not observed for Δ*hla*Δ*hlgABC*Δ*lukED*Δ*lukGH*Δ*pvl* (Figure 4A–C). Likewise, inoculation of human erythrocytes with USA300 mutants lacking *psm*α produced significantly less absorbance at 405 nm and LDH release relative to USA300 wt, while no significant differences were noted between erythrocytes inoculated with USA300 wt and Δ*hla*Δ*hlgABC*Δ*lukED*Δ*lukGH*Δ*pvl* (Figure 4E–G). To verify the importance of PSMα for hemolysis of human erythrocytes caused by USA300, we tested the hemolytic capacity of USA300Δ*psm*α complemented with a plasmid encoding *psm*α (USA300Δ*psm*α+comp). In both intoxication (Figure 5A) and co-culture assays (Figure 5B), complementation of USA300Δ*psm*α rescued hemolysis. To determine if PSMα is important for hemolysis of human erythrocytes by other strains of *S. aureus*, we measured hemolysis caused by an isogenic deletion mutant of *psm*α in the clinically relevant strain PFGE-type USA100 (USA100Δ*psm*α) compared to the parental wt. Parallel to prior results examining USA300, human erythrocytes that were intoxicated with extracellular factors produced by USA100Δ*psm*α (Figure 4D) or infected by this strain (Figure 4H) produced significantly less hemolysis relative to USA100 wt. Collectively, these results show that PSMα is the primary factor produced by *S. aureus* responsible for causing hemolysis of human erythrocytes.

## 3. Discussion

This investigation demonstrates that the PSMα peptides play a dominant role in causing hemolysis of human erythrocytes by two clinically prominent strains of MRSA: USA300 and USA100. This conclusion was initially indicated by results showing that the loss of Sae, a two-component system that upregulates Hla and the family of bicomponent leukocidins [34,35,36] but not PSMs [23,24], only has a minor impact on hemolysis, while loss of Agr, a two-component system that upregulates all cytotoxins including PSMs [21,22], abolishes hemolytic activity. Further analysis comparing hemolysis caused by USA300 that expressed all cytotoxins except PSMα to USA300 that only expressed the PSMs revealed that PSMα is the major factor causing hemolysis of human erythrocytes. The importance of PSMα was confirmed by repeating intoxication and co-culture assays using USA300 lacking PSMα that was complemented with a plasmid encoding this cytotoxin. Further, the loss of PSMα in a different clinically relevant MRSA strain, USA100, significantly reduced hemolytic activity. These findings are supported by previously published reports showing that purified PSMα peptides exhibit strong hemolytic activity against human erythrocytes [32] and that extracellular factors produced by isogenic deletion mutants of PSMα in *S. aureus* strains ST59 [37] or RJ-2 [38] have significantly reduced hemolytic activity against human erythrocytes.

Though we did not observe significant hemolytic activity caused by cytotoxins other than PSMα, others have shown that purified HlgAB, LukED, Hld, and PSMβ1 are also hemolytic against human erythrocytes [28,29,30,31,32]. Our results do not contradict these studies but instead indicate that PSMα produced during in vitro culture of *S. aureus* or during co-culture with human erythrocytes is more potent than other hemolysins. This may be due to higher levels of expression and/or a more rapid mechanism of action of PSMα that masks the influence of other cytotoxins. Others have shown that extracellular factors produced by the non-clinical *S. aureus* strain Newman cause hemolysis that is primarily mediated by HlgA [39]. However, Newman has a point mutation in the SaeS histidine kinase sensor that causes constitutive activation of the Sae two-component system and overexpression of HlgAB [40,41]. In contrast, this same study found hemolysis caused by USA300 was Agr-dependent but did not require HlgAB [39], congruent with findings in this report.

Others have demonstrated that hemolysin B (Hlb) exhibits synergistic activity with PSMs against a variety of mammalian erythrocytes, including human erythrocytes [32,38,42]. Notably, purified Hlb alone appears to have minimal activity against human erythrocytes in the absence of PSMs [32]. Though most strains of *S. aureus* isolated from animals produce Hlb, expression of this sphingomyelinase is deactivated in 87–96% of *S. aureus* strains found in humans. As the majority of prominent human clinical *S. aureus* isolates do not express Hlb, including USA300 and USA100, the hemolytic activity of this cytotoxin was not addressed in this study.

It has been reported that the *psm*α locus regulates expression of Hla [43], suggesting that reduced hemolytic activity of USA300Δpsmα may be due to decreased expression of Hla. However, we did not observe any significant difference in hemolysis caused by Δ*hla*Δ*hlgABC*Δ*lukED*Δ*lukGH*Δ*pvl* relative to USA300 wt. Further, the removal of *psm*α from Δ*hla*Δ*hlgABC*Δ*lukED*Δ*lukGH*Δ*pvl* resulted in a marked reduction in hemolytic activity. These findings correspond to published reports by others that show the loss of PSMα, but not Hla, significantly reduces hemolysis of human erythrocytes [37]. Taken together, these findings indicate that it is the loss of PSMα and not reduced expression of Hla in mutants lacking psmα that is responsible for decreased hemolytic activity of *S. aureus* deletion mutants of *psm*α.

It has been demonstrated that hemolysis caused by HlgAB and LukED requires expression of the DARC receptor by human erythrocytes [28]. This chemokine receptor is used by the malarial pathogens *Plasmodium vivax* and *Plasmodium knowlesi* to invade erythrocytes [44,45]. Consequently, some individuals of African descent do not express DARC to provide protection against malarial pathogenesis. Though a small subset of the human population is negative for DARC, we did not detect differences in DARC expression on erythrocytes from any of the donors used in this investigation, indicating that PSMα is the dominant hemolytic factor produced by *S. aureus* against erythrocytes susceptible to HlgAB and LukED.

Expression of *S. aureus* cytotoxins during infection is thought to be largely driven by host-associated environmental signals recognized by two-component regulatory systems, and studies examining hemolysis caused by purified proteins or *S. aureus* cultured in vitro may not accurately represent hemolytic activity in vivo. Though our investigation examined hemolysis of erythrocytes infected by live *S. aureus* over time, these conditions lack some of the environmental cues encountered in the host that may be needed to elucidate the importance of other cytotoxins. For example, expression of the *hlgABC* operon by USA300 is highly upregulated following exposure to whole human blood [46], and additional host-associated environmental triggers may need to be incorporated into hemolysis assays to elucidate a more prominent role for HlgAB and other *S. aureus* cytotoxins.

## 4. Materials and Methods

### 4.1. Bacterial Strains and Culture Conditions

*S. aureus* was cultured in tryptic soy broth (TSB; EMD Millipore, St. Louis, MO, USA) in a shaking incubator at 250 rpm and 37 °C. A 1:100 dilution of overnight cultures grown for 12–15 h in TSB was used to start subcultures in a 14 mL culture tube containing 5 mL TSB (1:100 dilution). *S. aureus* strains identified as PFGE-type USA300 strain Lac and PFGE-type USA100 strain 252 used in this study have been described previously [47,48]. *S. aureus* mutants used in this study (Table 1) were from previous investigations or generated using published protocols [34,35,49,50,51,52,53] with primers listed in Table 2. Complementary plasmids generated in this investigation used PCR amplification with primers and restriction enzyme sites listed in Table 2 to clone cytotoxin genes into pRB473, as previously described [35,53].

### 4.2. Human Erythrocyte Preparation

Human erythrocytes were prepared from freshly drawn heparinized venous blood from healthy donors following a standard IRB-approved protocol (protocol number JVK040821, Institutional Review Board, Montana State University) with written informed consent. Freshly drawn blood was resuspended in ten times the blood volume of PBS and centrifuged (1000× *g*, 5 min). Supernatant was removed, and red blood cells were washed twice more with PBS, then immediately used for preparation of human blood plates, supernatant intoxication assays, or *S. aureus* co-culture assays as described below.

### 4.3. Blood Agar Hemolysis Assays

*S. aureus* was subcultured to mid-exponential growth phase and dilutions plated on tryptic soy agar with 3% human erythrocytes or 5% sheep erythrocytes. Plates were incubated at 37 °C with 5% CO_2_ for 24 h. Following incubation, images of plates were taken and diameter of hemolysis from three replicate colonies was measured.

### 4.4. Intoxication Assays

Washed erythrocytes were resuspended to a final dilution of 1:200 in DPBS and 100 μL aliquoted into wells of a 96-well plate with clear flat-bottom and black side-walled wells. *S. aureus* strains subcultured for six hours unless otherwise indicated in TSB were centrifuged (5000× *g* for 5 min), and 100 μL of collected supernatant was immediately added to wells containing erythrocytes. Samples were agitated for 10 s, and turbidity was assessed by measuring absorbance at 630 nm every minute for 60 min at 37 °C using an Epoch2 microplate spectrometer or Cytation5 microplate spectrometer (Agilent Bio-Tek, Winooski, VT, USA). Lactate dehydrogenase (LDH) release was measured after 40 min of intoxication using a Cytotoxicity Detection KitPLUS (Roche, Switzerland) following the manufacturer’s protocol.

### 4.5. Co-Culture Assays

Washed erythrocytes were resuspended to a final dilution of 1:200 with RPMI. Subcultured *S. aureus* was harvested at mid-exponential growth (2 × 10^8^ CFU/mL) by centrifugation (5000× *g* for 5 min) and washed twice with DPBS. *S. aureus* was resuspended in 1 mL of RPMI and then combined with 5 mL of washed erythrocytes in 15 mL culture tubes for a final concentration of 3.3 × 10^7^ CFU/mL unless otherwise indicated. Samples were incubated at 37 °C with shaking at 250 rpm. At indicated time points, 200 μL of infected erythrocytes were centrifuged (5000× *g*, 5 min) and 175 μL of supernatant transferred to a 96-well plate with clear flat-bottom and black side-walled wells. To assess heme released from lysed erythrocytes, absorbance at 405 nm was measured at indicated time points. Lactate dehydrogenase (LDH) release was measured at six hours post-inoculation, as described above.

## Figures and Tables

**Table 1:** Strains used in this study.

Strain	Reference
USA300	[47]
USA300Δ*hla*	[52]
USA300Δ*agrABCD*	[53]
USA300Δ*saePQRS*	[53]
USA300Δ*hla*Δ*hlgABC*Δ*lukED*Δ*lukGH*Δ*psm*αΔ*pvl*	[53]
USA300Δ*hlgABC*Δ*lukED*Δ*lukGH*Δ*pvl*	[53]
USA300Δ*hla*Δ*psm*α	[53]
USA300Δ*psm*α	this study
USA300Δ*hla*Δ*hlgABC*Δ*lukED*Δ*lukGH*Δ*pvl*	this study
USA100	[48]
USA100Δ*psm*α	this study
USA300 pRB473	this study
USA300Δ*psm*α pRB473	this study
USA300Δ*psm*α pRB473-*psm*α	this study

**Table 2:** Primers used in this study.

Primer	Sequence
*psm*α-Top_fwd	5′-GGG GAC AAG TTT GTA CAA AAA AGC AGG CGT CGT CTA CCT TTC CAT GC-3′
*psm*α-SphI-Top_rvs	5′-GGT GGT GCA TGC CTC AGG CCA CTA TAC CAA TAG-3′
*psm*α-SphI-Bot_fwd	5′-GGT GGT GCA TGC CAG CGA TGA TAC CCA TTA AGA TTA CC-3′
*psm*α-Bot_rvs	5′-GGG GAC CAC TTT GTA CAA GAA AGC TGG GTC GAA TGC AAG CCA ACC AC-3′
*hla*-Top_fwd	5′-GGG GAC AAG TTT GTA CAA AAA AGC AGG CGA AGT CCA TAC AAA ATC CGC ATC-3′
*hla*-BamHI-Top_rvs	5′-GGT GGT GGA TCC CTA TCT ACT TGA TTT GCT TTC CTG AC-3′
*hla*-BamHI-Bot_fwd	5′-GGT GGT GGA TCC CAA TTT CGA GGG TTA GTC AAA GTT G-3′
*hla*-Bot_rvs	5′-GGG GAC CAC TTT GTA CAA GAA AGC TGG GTG CAA TAC TTT ATT GTC CCA TGA TTA GTG-3′
*psm*α-comp-SacI_fwd	5′-CCA CCA GAG CTC CTA GAC GAG ACC TAA CGT G-3′
*psm*α-comp-BamHI_rvs	5′-GGT GGT GGA TCC CTA TTG GTA TAG TGG CCT GAG-3′

## Data Availability

The original contributions presented in this study are included in the article. Further inquiries can be directed to the corresponding author.
